# Nuclear Factor-kappaB in Autoimmunity: Man and Mouse

**DOI:** 10.3389/fimmu.2018.00613

**Published:** 2018-04-09

**Authors:** Bahar Miraghazadeh, Matthew C. Cook

**Affiliations:** ^1^Centre for Personalised Immunology, John Curtin School of Medical Research, Australian National University, Acton, ACT, Australia; ^2^Translational Research Unit, Canberra Hospital, Acton, ACT, Australia; ^3^Department of Immunology, Canberra Hospital, Acton, ACT, Australia

**Keywords:** NF-κB, autoimmunity, self-tolerance, thymic development, mutation

## Abstract

NF-κB (nuclear factor-kappa B) is a transcription complex crucial for host defense mediated by innate and adaptive immunity, where canonical NF-κB signaling, mediated by nuclear translocation of RelA, c-Rel, and p50, is important for immune cell activation, differentiation, and survival. Non-canonical signaling mediated by nuclear translocation of p52 and RelB contributes to lymphocyte maturation and survival and is also crucial for lymphoid organogenesis. We outline NF-κB signaling and regulation, then summarize important molecular contributions of NF-κB to mechanisms of self-tolerance. We relate these mechanisms to autoimmune phenotypes described in what is now a substantial catalog of immune defects conferred by mutations in NF-κB pathways in mouse models. Finally, we describe Mendelian autoimmune syndromes arising from human NF-κB mutations, and speculate on implications for understanding sporadic autoimmune disease.

## Introduction

NF-κB is family of proteins that mediate transcriptional regulation crucial to many biological functions (1). NF-κB was discovered and named for its action in regulating κ light chain expression in B cells. Remarkably, however, as elucidation of *Drosophila* immunity has demonstrated, NF-κB and its orthologs are highly conserved regulators of signaling and transcription crucial for cellular and humoral host defense even in organisms that lack adaptive immunity (2). In mammals, the actions of NF-κB are complex and extensive, and encompass activation, proliferation in cells of the innate and adaptive immune system, and organogenesis of lymphoid tissue and its microarchitecture (3–5).

**Figure 1:**
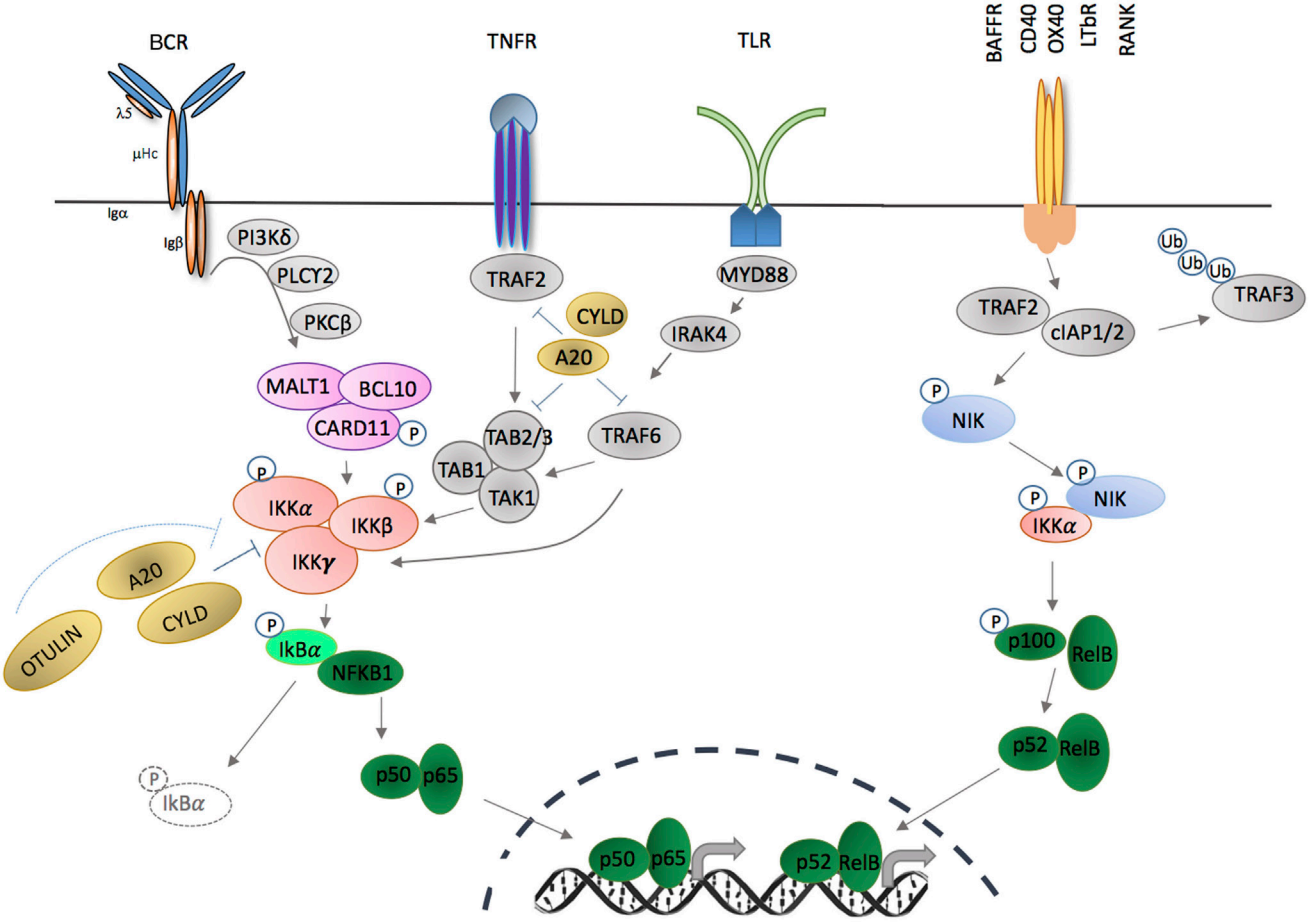
Activation of canonical and non-canonical NF-κB signaling pathways through membrane-bound extracellular ligands. TNFR and toll-like receptor (TLR) family members, as well as antigen receptors activate the canonical pathway; and regulation of B cell activating factor receptor (BAFFR), CD40, OX40, LTβR, and receptor activator of nuclear factor kappa-B (RANK) activate the non-canonical pathway. Triggering of canonical pathway results in activation of p50/p65 (RelA), while the non-canonical pathway signaling leads to activation of p52/RelB complexes. Both pathways require phosphorylation and activation of inhibitor of kappa B kinase (IKK) subunit(s) in order to release NF-κB molecules that are sequestered by an inhibitor, e.g., IκBα or p100. Phosphorylation and ubiquitination of the inhibitors by IKKs release NF-κB that translocate into nucleus in the forms of homodimers or heterodimers complexes and bind to the κB site of their target genes.

Under normal circumstances, NF-κB proteins are latent in the cytoplasm, poised for rapid responses after their inhibition is temporarily removed. Uninhibited NF-κB molecules then shuttle between nucleus and cytoplasm as transcriptionally active homo- and heterodimers (Figure 1). In addition to this fundamental inhibitory constraint, many other negative regulatory loops exist to either prevent, dampen, or terminate NF-κB signaling, including sequestration in multi-molecular complexes, posttranscriptional regulation, and posttranslational modifications of proteins by phosphorylation and ubiquitination (of various forms). Furthermore, many components of NF-κB, including both positive and negative regulators, are under transcriptional regulation by NF-κB itself.

Despite this complex regulatory network, specific defects in individual molecules within the NF-κB pathway have been shown to disrupt cellular homeostasis, and immune pathology is an important consequence (1, 6). In this review, we will concentrate on how NF-κB contributes to immunological self-tolerance, and how defects in NF-κB contribute to autoimmune disease. Defects in NF-κB have also been shown to cause immune deficiency and autoinflammatory diseases, and somatic mutations are frequent drivers of lymphoid malignancy, for which authoritative reviews are available (7, 8). As will be discussed here, however, it is notable that in some cases, a single mutation confers both autoimmunity and immune deficiency, reflecting the complex regulatory actions of NF-κB.

## Outline of Normal NF-κB Signaling

The NF-κB family of transcription factors form hetero- and homodimers that regulate transcription by binding to a palindromic DNA sequence, κB (1), located within promoters and enhancers of a large number of genes (9, 10). In vertebrates, there are five NF-κB family members, RelA, c-rel, RelB, NF-κB1, and NF-κB2. N-terminal Rel-homology domains (RHD, from v-Rel, reticuloendotheliosis viral oncogene homolog) are common to all and mediate κB binding and interactions with other proteins, including inhibitor of kappa B (IκB) (see below) (10–12).

NF-κB proteins are classified in two groups according to structure and function. p105 (NF-κB1) and p100 (NF-κB2) are precursor proteins that undergo partial proteolysis to remove their C-terminal ankyrin repeats, yielding p50 and p52, respectively. p50 and p52 lack transactivation domains (TAD) unless heterodimerized with Rel or coactivator non-Rel proteins (13). By contrast, RelA (p65), RelB, and c-Rel are active in the absence of proteolysis because they contain TAD that positively regulate expression of target genes (14, 15).

Differences in transcriptional activity of NF-κB dimers helps explain the plasticity of responses to both quantitative and qualitative variation in cell stimulation (16). p50/65 heterodimers are near ubiquitous, and positively regulate NF-κB target genes (10). By contrast p50 homodimers repress TNF-α transcription in response to lipopolysaccharide (LPS) (17–19). Homodimers of p50 are abundant in resting T cells, but their expression is reduced after antigenic receptor ligation (20), when p50/p65 become abundant in cell nuclei, reversing the NF-κB-dependent suppression of the target genes, i.e., IL-2 or IL-6 and iNOS in response to LPS (21). RelB does not homodimerise, but confers transcriptional activity when complexed with p52 or p50 (22). RelB constitutively localizes to the nucleus, but binding may be inhibited by association with p100 (23–25). Under some circumstances, RelB represses NF-κB activity by forming RelA/RelB heterodimers that fail to bind DNA and sequesters RelA (9, 26, 27). Similarly to RelB, c-Rel is expressed in lymphoid tissues, and both c-Rel homodimers and c-Rel/p50 heterodimers are detected predominantly in hematopoietic cells. c-Rel homodimers and c-Rel/p65 are essential for B and T cells survival and effector cell function (28–30).

## Activation of NF-κB in Immunity

In the absence of specific signals, NF-κB is maintained in latent form bound to IκB in the cytoplasm. The IκB family includes IκBα, IκBβ, IκBε, BCL-3, IκBζ, IκBNS, as well as unprocessed p100 and p105, which are all characterized by multiple ankyrin repeats (31). In addition to their IκB function, so-called atypical IκBs (IκBζ, IκB-NS, and Bcl-3) have intrinsic nuclear localization propensity where they bind preferentially to p50 (Bcl-3, IκBζ) and p52 (Bcl-3), which under different conditions can either promote or terminate NF-κB binding to DNA (32, 33). This mechanism has been well characterized for Bcl-3, which has a TAD and can act as a coactivator when associated with p50 or p52 homodimers (34, 35), or by facilitating replacement of transcriptionally inactive p50 homodimers with transcriptionally active heterodimers (36, 37). On the other hand, Bcl-3 can also function as a repressor by stabilizing p50 homodimers on κB site of the target genes. For example, Bcl-3 has been reported to mediate LPS tolerance in macrophages by stabilizing p50 homodimer on the *TNF* promoter (38–40).

NF-κB members are liberated by the action of the IκB kinase complex [IKKα, IKKβ and NF-κB essential modulator (NEMO), encoded by *IKBKA, IKBKB* and *IKBKG*], which leads to temporary degradation of IκBs (Figure 1). IKKγ (NEMO) is a regulatory subunit that does not have intrinsic catalytic activity but is important for kinase activation of IKKα and IKKβ (41). IKKα and IKKβ are serine/threonine kinases that share an N-terminal kinase domain and are responsible for phosphorylating several members of the IκB family (42, 43). Serine phosphorylation of IKKα and IKKβ [serines 177 and 181 for IKKβ; serines 176 and 180 for IKKα (44, 45)] results in conformational change and kinase activation. Activated IKKβ operates within the inhibitor of kappa B kinase (IKK) complex to phosphorylate IκBα, leading to K48-linked ubiquitination and proteosomal degradation, which releases NF-κB factors for nuclear translocation (46, 47). Loss of IKKβ results in significant reduction of NF-κB activity in response to TNF-α and IL-1α, a defect that cannot be completely compensated for by IKKα. As result, *Ikbkb* deficiency confers a mouse phenotype similar to *Rela* deficiency (48, 49) (Table 1).

**Table 1:** Summary of autoimmune phenotypes in mice with genetic manipulation of NF-κB.

Gene	Protein	Mutation	B cell phenotype	Regulatory T cell (Treg) phenotype	Autoimmunity or inflammation	Reference
*Map3k7*	TAK1	Conditional deletion (T cell)		Treg deficient	Severe late colitis	(50)
	
		Conditional deletion (Tregs)		Peripheral Treg deficient	Mild autoimmunity, splenomegaly and lymphadenopathy, renal hemorrhage	(51)
	
		Deletion	Reduced B cells	Reduced T cells	Liver failure, ascites, jaundice	(52)

*Card11*	CARD11	L298Q [*N*-ethyl-*N*-nitrosourea (ENU)]	Absent B1 cells, abnormal B cell maturation, defective B cell responses	Treg deficient	Dermatitis with mast cell and eosinophil infiltrates. Concomitant defect in Tregs and conventional T cells	(53, 54)
	
		L525Q (ENU)	B1 cell deficiency, impaired B cell proliferation	Thymic Treg deficiency, reduced peripheral Tregs	Late onset dermatitis	(55)
	
		Deletion		Natural regulatory T cell deficiency, Treg precursor deficiency	No autoimmune due to concomitant defect in T cell function	(56)

*Malt1*	MALT1	C472A	Impaired B1 and marginal zone (MZ) B cells development	Defect in thymic Tregs with reduction in peripheral Tregs	Spontaneous autoimmune gastritis	(57)
	
		Deletion	Impaired follicular, B1, and MZ B cells development	Defect in thymic Tregs with reduced peripheral Tregs	Resistance to experimental autoimmune encephalomyelitis (EAE)	(58–60)

*Bcl10*	Bcl-10	Deletion	Defect in follicular, B1, and MZ B cell	Treg deficiency	Increased susceptibility to bacterial sepsis	(61–63)

*Ikbka*	IKKα	Conditional deletion (CD4)		Treg reduction	Increased susceptibility to colitis	(64)
	
		Deletion			Severe skin and skeletal abnormalities	(65, 66)

*Ikbkb*	IKKβ	Conditional deletion (T cell)	Defect in memory B cells and reduced germinal center (GC) B cells	Treg deficient	No autoimmunity or inflammation	(67)

*Ikbkg*	IKKϒ	Conditional deletion		Treg deficiency	Skin inflammation, epidermal granulocytic infiltration, liver apoptosis	(67, 68)

*Map3k14*	NF-κB-inducing kinase	G855R (*aly*)	Reduced B cells, defective GC formation	Treg deficiency	Spontaneous inflammation	(69–72)
	
		Deletion		Treg deficiency	Multi-organ inflammation	(73)

*Nfkbia*	IKBα	Conditional altered κB enhancer		Defect in T cell development and activation; low naive T cell, high memory T cells; Treg defect independent of IKBa-mediated feedback regulation of NF-κB	Sjogren’s syndrome	(74)
	
		Deletion			Anemia; thymic atrophy; small spleen and liver	(75)

*Nfkb1*	p50	Deletion	Defect in terminal B cell differentiation, mature B cell apoptosis, reduced transitional and MZ B cells		Sepsis in response to selective pathogens; chronic inflammation; premature aging and premature death	(76–80)
	
		Deletion	Defect in T1 to T2 transition, and in MZ B cells; reduced GC B cells in young mice, absent GC structure		Lymphoproliferative disease and multi-organ autoimmunity	(81)

*Nfkb2*	p52	Deletion	Defect in follicular MZ and MZ B cells; impaired GC formation			(82, 83)

*Rela*	RelA	Conditional deletion (Treg)		Reduced Treg precursors, decreased Tregs with impaired function	Severe multi-organ inflammation, lymphoproliferation	(84, 85)

*Relb*	RelB	Deletion	Defect GC formation	Mild T cell depletion in spleen and lymph nodes	Multi-organ inflammation	(86, 87)

*Rel*	c-Rel	Conditional deletion (Treg)		Reduced Treg precursors, decreased Tregs with impaired function	Late onset mild inflammation	(85, 88)
	
		Deletion	Defect B cell proliferation	Defect T cell proliferation		(28)

*Bcl-3*	Bcl-3	Conditional deletion (T cell)		Impaired Th1 differentiation, fewer pathogenic Th17-like cells	Resistance to colitis and EAE	(89)
	
		Deletion	Increased MZ B cells, reduced follicular transitional B cells, defect GC formation		Susceptibility to *T. gondii*, multi-organ inflammation	(90, 91)

*Nfkbid*	IKBNS	Deletion		Treg deficiency	Resistance to Th17-dependent EAE	(92, 93)

*Nfkbiz*	IKBƺ	Deletion		No effect on Treg, Impaired CD4 T cells and Th17 development	Resistance to TNFα- IL-17A- inducible psoriasis like skin inflammation, atopic dermatitis, resistance to EAE	(94, 95) (96)

*Otulin*	OTULIN	Conditional deletion (myeloid)	B cell hyperactivity		Autoimmunity, multi-organ inflammation	(97)
	
		Conditional deletion (immune cells)			Multi-organ inflammation	(97)

*Tnfaip3*	A20	Conditional deletion (B cells)	Increased B cell proliferation and activation, defect MZ B and B1 cells differentiation	Treg expansion	Autoimmunity	(98)
	
		Conditional deletion (dendrite cells)	Increased B cell activation and differentiation	Increased T cell activation and expansion	SLE-like phenotype; IBD-like phenotype	(99, 100)

*Cyld*	CYLD	Deletion	Increased MZ B cells in aged mice	Defect T cell development, hyper responsive to anti-CD3, -CD28	Spontaneous intestinal inflammation	(101–103)
	
		CYLD^x7/8^ naturally overexpressed	Increased mature B cell, enhanced B cells survival	Enhanced Treg, defect Treg function and survival	Large spleen and lymph nodes	(104, 105)

## Canonical Pathway Stimuli

The canonical NF-κB pathway includes NFκB1 (p105), RelA (p65), and c-Rel and is activated by many ligands, including those that engage members of the tumor necrosis factor receptor superfamily, toll-like receptors (TLR), interleukin 1 receptor, and T and B cell antigen receptors (Figure 1). Different receptor-proximal signaling cascades connect these receptors to the IKK complex. TLR ligation activates a complex composed of TAK1, TAB 1, TAB 2, which phosphorylates IKKβ (106, 107). Activation of TNF receptor leads to interaction of series of adaptor proteins that contains TRAF-binding domains (108, 109). TRAF2 recruits the E3 ubiquitin ligases, cIAP1/2, which are necessary for IKK activation *via* recruitment of linear ubiquitination assembly complex (LUBAC) (directly) and TAK1 (indirectly) (110, 111). Ligation of B and T cell antigen receptors leads to phosphorylation of CARD11 by protein kinase C and assembly of the CARD/BCL-10/MALT1 (CBM) complex.

## Modification of Canonical Signaling

Posttranslational regulation by ubiquitination is crucial for NF-κB regulation. A series of ubiquitinating and deubiquitinating enzymes are known that both activate and modify NF-κB transcriptional regulation (112). The best characterized negative regulators of NF-κB are deubiquitinase (DUB) A20 (encoded by *TNFAIP3*), CYLD, and OTULIN (113–116).

TNFAIP3 is upregulated in response to TNF receptor and TLR ligation, and A20 negatively controls NF-κB-dependent gene expression (Figure 1) (117). A20 removes or modifies K63 polyubiquitin from several substrates within NF-κB signaling, including TRAF6, NEMO, MALT1, and TNFR1 (118–120). In addition to its deubiquitin domain in the N-terminus, A20 also contains seven zinc finger domains in the C-terminus with E3 ligase functions, enabling ubiquitin-editing. Thus, A20 replaces K63- with K48-polyubiquitin chains of RIPK1, flagging it for proteasomal degradation (99, 113). The importance of A20 in modifying NF-κB activity and immune responses was confirmed in A20-deficient mice that developed profound inflammation and cachexia, and died prematurely (117) (Table 1), and cell-specific deletion of A20 in myeloid cells, dendritic cells (DCs) or B cells, results in autoimmune phenotypes including polyarthritis and enteritis (Table 1) (121–124). Loss of catalytic activity might not account for this pro-inflammatory action, however, as specific introduction of a deubiquitination domain inactivating mutation resulted in a much less prominent inflammatory phenotype (125).

NF-κB is also modified by addition of methionine (M)1-linked linear ubiquitin chains by the LUBAC that consists of HOIL-1L (*RBCK1*), HOIP (*RNF31*), and SHARPIN (112). LUBAC promotes canonical signaling in part through direct binding of the E3 ligase activity of the ring domains of HOIP to drive linear ubiquitination of NEMO and RIP1 (126), which promotes IKKβ phosphorylation and activation through TAK1 (107, 127, 128). CYLD modifies canonical signaling by removing K63 and M1-polyubiquitin chains from NF-κB signaling proteins, including TRAF2, TRADD, NEMO, and TAK1 (Figure 1). CYLD appears to be particularly important in promoting necroptosis *via* RIPK1. CYLD deficiency results in several non-immunological phenotypes, but also inflammatory bowel disease, defects in thymic medullary epithelial cells (see below), and increased B cell activity (Table 1) (105, 129). OTULIN specifically hydrolyzes M1-polyubiquitins. In mice, germline deficiency of *Otulin* is lethal, but conditional myeloid deficiency results in profound sterile inflammation (Table 1) (97). Interestingly, after caspase-mediated cleavage during apoptosis, the N-terminal fragment of HOIP also binds to the DUBs OTULIN and CYLD, which are down-regulators of LUBAC-mediated ubiquitination, providing a further regulatory feedback loop (130).

## Non-Canonical Signaling

NFκB2 (p100) and RelB participate in the non-canonical NF-κB pathway (Figure 1). Unlike the canonical pathway, which is poised for rapid response, the non-canonical pathway depends on constant protein synthesis (131). This might explain the preferential action of the non-canonical pathway in cellular homeostasis and organogenesis, whereas the canonical pathway mediates acute inflammatory responses and immune activation.

Non-canonical NF-κB activation is stimulated by a relatively small number of ligands, including lymphotoxin, CD40, B cell activating factor (BAFF), and receptor activator of NF-κB (RANK). In resting cells, TRAF2 mediates ubiquitination of NF-κB-inducing kinase (NIK) in association with TRAF3 and cIAP1/2. NIK is maintained in limiting concentrations by rapid degradation after phosphorylation by TRAF6, but after receptor ligation, TRAF2-induced proteolysis and degradation of TRAF3 and TRAF6 leads to NIK accumulation, which phosphorylates IKKα (132, 133). pIKKα is crucial for phosphorylation of p100, leading to proteosomal processing to p52 (134–140).

Non-canonical signaling activates complex regulatory loops because p100 exerts IκB activity by associating with p52, p65 (RelA), c-Rel, and RelB. Interestingly, and by contrast with the IκB activity of the canonical counterpart p105, p52 is formed from recently synthesized p100. Elegant experiments have revealed that the balance between p100 activation and NIK degradation is maintained within a kappaBsome, which is a complex of NIK-IKKα-p100 and RelB. Almost all p100 is maintained within the kappaBsome, since free cytoplasmic p100 undergoes complete degradation, whereas processing from within the kappaBsome yields p52 (141). RelB promotes formation of the kappaBsome, and competes with IKKα–NIK complexes to inhibit p100 activation and processing. In addition to promoting p100 processing, IKKα serves to limit activity and abundance of NIK within the complex, and p100 inhibits NIK degradation. RelB dimerizes with both p52 and p100 in the complex to permit the proper processing of p100. In the absence of RelB, p100 undergoes complete degradation (141, 142).

## Transcriptional Regulation by NF-κB

NF-κB lacks the enzymatic activity to directly regulate transcriptional responses, and this is achieved through binding to transcriptional co-regulators including histone acetyltransferases (HATs) and histone deacetylases (HDACs). For instance, acetylation of p65 at different lysines by HATs (and the associated binding proteins) promotes its activity, while the co-repressor, HDACs, can deacetylate important sites on p65 and reverse the effect (143). NF-κB recruit these co-regulators to the enhancer region of the target genes, leading to conformational changes in chromatin to make genes accessible for transcriptional machinery (143–145).

Binding to HATs is also controlled partially by posttranscriptional modifications (PTMs). For example, kinases including PKA, MSK1, and MSK2 phosphorylate p65 at Serine 276, which is crucial for the interaction of p65 with HATs (21, 26, 146, 147). Interestingly, this amino acid substitution only affects expression of a subset of the genes, which suggests other PTMs might contribute to p65-dependent chromatin remodeling (148). One example is Akirin2, which binds to HATs and IκBζ and facilitates recruitment of other co-regulators to p50 (149).

## NF-κB and Self-Tolerance

Canonical and non-canonical NF-κB pathways are crucial for T and B cell activation, regulation of inflammatory effector responses, antigen presentation and regulation of tissue-specific cellular targets of immunity. We will concentrate our attention on mechanisms that regulate lymphocyte self-tolerance, since these are most relevant to autoimmunity. These actions can be divided into those that act intrinsically within lymphocytes, and those that are mediated by lymphocyte extrinsic actions, particularly for thymic selection.

## Thymic Development

The thymus is derived from the endoderm of the third pharyngeal pouch. Mature thymic epithelium is made up of cortical thymic epithelial cells (cTEC) and medullary thymic epithelial cells (mTEC). Unlike other epithelia, thymic epithelium lacks apical polarity, and cells are dispersed in a three-dimensional reticular meshwork (150). The two best understood mechanisms of central T cell tolerance are negative selection of self-reactive conventional T cells (Tconv), and selection of regulatory T cells (Tregs). Both mechanisms depend on maturation of mTECs, and expression of autoimmune regulator (Aire), which in turn regulates expression of tissue specific antigens (TSA) (151–153).

Both mTECs and cTECs have been postulated to arise from CD205^+^ β5t^+^ and CD45^−^ bipotential precursors (154). SSEA-1^+^ Claudin3/4^+^ expression identifies a self-renewing population of CD80^lo^ MHCII^lo^ mTEC precursors (155), which exhibit some epithelial stem cells characteristics. cTECs are characterized by epithelial cellular adhesion molecule (Ep-CAM) β5t, CD205, and Ly51 expression (156). Mature mTECs are UAE-1^+^ Ly51^−^, MHCII^hi^, CD80^hi^, CD40^+^, and express Aire. mTECs are responsible for maintenance of T cell tolerance *via* negative selection of αβ T cells (157, 158), and positive selection of FOXP3^+^ Tregs, by virtue of promiscuous expression of tissue specific antigens (TSA) (154, 159, 160). Terminally differentiated mTECs (which form Hassall’s corpuscles) are marked by loss of AIRE, MHCII, and CD80, acquisition of desmogleins 1 and 3, and of involucrin, a global marker of epithelial terminal differentiation (161).

## NF-κB and Development of Thymic Epithelium

Thymus development depends on an unusual hematopoietic–epithelial interplay between epithelial cell precursors and nascent lymphocytes (162). Interestingly, lymphocytes exert a similar influence on development of the specialized epithelium overlaying Peyer’s patches (M cells) (163). In both thymus and Peyer’s patches, epithelial development is at least partially dependent on NF-κB activation by RANKL and other ligands, delivered by lymphocytes (164–166). Selection of developing T cells depends on lymphocyte–epithelial interactions within the thymus, and interactions between immature T cells and thymic epithelium regulates T cell development by negative selection of self-reactive Tconv, and positive selection of regulatory Tregs (157, 161, 167).

Signaling *via* the non-canonical NF-κB pathway appears to regulate mTEC maturation and the size of the mTEC compartment, but not necessarily specification of the mTEC lineage. This action hinges on NF-κB activation by ligation of TNF receptor superfamily members RANK, CD40, and LTβR (86, 166, 168). Single positive thymocytes, γδ T cells, invariant NK cells, and lymphoid tissue inducer cells are all sources of RANKL and CD40L (165, 166). Fate-mapping studies have provided more information on the sequential actions of non-canonical stimuli for development and maintenance of mTECs. Thus, RANK operates after mTEC precursors have differentiated and coincides with onset of RelB expression (169). RelB appears to act cell-intrinsically to determine mTEC numbers (86, 168), while RelB expression mediates the non-canonical pathway, it is also modified by RelA and c-Rel, and therefore, mTEC development can be influenced by canonical NF-κB signaling (170). Late mTEC differentiation is also disrupted by altered CYLD function, suggesting that regulation of RANK signaling *via* TRAF6 deubiqutination might be important during late mTEC development (171), possibly by regulating RelB induction by the canonical NF-κB pathway.

As well as RelB deficiency, deficiencies of NIK, IKKα, LTβR, NF-κB2 (p100), and Bcl-3 result in mTEC deficiencies of varying severity (172–174). Interestingly, the thymic defect observed with *Nfkb2* deficiency is less marked than that observed with *Relb* deficiency (175). mTEC-specific deletion of LTβR results in disordered thymic architecture, but not altered negative selection, although negative selection is defective when LTβR is deleted from thymic DCs (159). Indeed, distinguishing the actions of defects in mTECs from those in DCs requires construction of chimeras in which the mutation is confined to either hematopoietic or stromal compartments, which has not yet been performed for all mutations.

Bcl-3 action appears to be redundant with NF-κB2 for mTEC development (176). Thus, mice doubly deficient for *Bcl3* and *Nfkb2* lack mTECs, thymic Aire expression, and some thymic DCs, and they develop severe organ-specific autoimmunity. NIK is crucial for non-canonical NF-κB activation, but the mechanism of Bcl-3 is less obvious. While Bcl-3 is an atypical IκB, it does not regulate RelA or c-Rel. Uncertainties regarding the mechanism of Bcl-3 notwithstanding, there is solid empirical evidence for a crucial cell-intrinsic action of non-canonical signaling for maintaining mTECs, and for central T cell tolerance. In addition, several findings suggest that these actions are modified by regulation of RelB expression and activation *via* the canonical pathway.

## NF-κB Defects Confer Susceptibility to Tissue-Specific Autoimmunity

Deficiency of Aire leads to widespread tissue-specific inflammation in man and mouse as a result of impaired expression of TSA by mTECs (158, 177, 178). This discovery represented a landmark in autoimmunity research, providing empirical evidence that negative selection mediated by antigen expression in mTECs is a bulwark against autoimmune disease (160). NF-κB signaling is important for Aire expression independently of mTEC development and homeostasis. The conserved non-coding sequence 1 (CNS1) located 3′ to the Aire transcription start site is an Aire enhancer, and contains two NF-κB-binding motifs, one with a preference for RelA and c-Rel, and the other for RelB (179, 180). There is compelling evidence for the interaction of RelA with CNS1, implicating the canonical NF-κB pathway in Aire expression. CD40 and RANK ligation appears to be important for Aire induction by NF-κB, whereas Aire expression is independent of LTβR (181).

More recently, FEZF2 was also identified as a transcriptional regulator of TSA expression in mTECs. NF-κB has been implicated in *Fezf2* expression, although the relative importance of LTβR and RANKL remains controversial (159, 182). *Fezf2* deficiency results in a pattern of organ-specific autoimmunity targeted at lung, kidney, liver, and intestine, but not retina or pancreas, which is observed in Aire deficiency (182). PRDM1 is the most recent addition to transcriptional regulators of TSA in mTECs, and while the regulation of expression PRDM1 in mTECs is unclear, it is known to be induced by RANKL-mediated NF-κB signaling in other tissues (183, 184).

As might be predicted from its crucial action on mTECs, *Relb* deficiency results in multi-organ inflammation, indicating that non-canonical signaling acting to regulate mTEC development is crucial for maintaining self-tolerance and prevention of organ-specific autoimmunity (86, 168). The autoimmune phenotypes of *Relb* mutants and related strains are summarized in Table 1. The severity of the autoimmune phenotype conferred by *Relb* deficiency may reflect its importance not only for mTEC development but also on Aire expression. Mice deficient in NIK (*Map3k14^−/−^*) also develop autoimmunity, providing additional evidence that the non-canonical pathway regulates self-tolerance (70, 71). Similarly, autoimmune phenotypes have been induced by deletion of CD40L, RANKL, or LTβR, the ligands crucial for inducing non-canonical signaling for mTEC maturation and maintenance (166, 185, 186).

Mice rendered deficient in *Nfkb2* also display modest defects in central tolerance and an autoimmune phenotype of inflammatory infiltrates in lungs, liver, kidneys, and pancreas, together with autoantibodies, thus resembling the phenotype of Aire deficiency (175). In this case, reciprocal bone marrow chimeras confirmed the defect was in the radio-resistant stroma, and further analysis revealed a numerical defect in Ep-Cam^+^ (G8.8) and *Ulex europaeus* agglutinin 1^+^ mTECs, a cell-for-cell defect in LTβR-induction of Aire, and a reduction in expression of Aire-dependent TSA. No numerical or functional Treg defect was observed. Interestingly, and by contrast with complete NF-κB2 deficiency, specific deficiency of p52 conferred only a T-cell dependent antibody response with no overt autoimmune phenotype (83, 187, 188).

## NF-κB and Treg Selection

NF-κB appears to be important for maintaining Treg function *via* mTEC development, thymic dendritic cell development, and T-cell-intrinsic actions (Table 1). TCR ligation and co-stimulation, particularly *via* CD28, activates the canonical NF-κB pathway, which appears to be important for FoxP3 induction (189). Engagement of TCR and CD28 activates the CBM complex, *via* the upstream and mediators, TAK1 and PKCθ. Deficiency in each of these signaling components alone also results in Treg deficiency (54–56, 190, 191). Activation of the canonical pathway induces c-Rel, which binds the conserved non-coding sequence (CNS)-3 enhancer of FoxP3 to promote its expression (192, 193). Thus, c-Rel deficiency results in a similar Treg deficiency, while Tregs are preserved in the face of p50 deficiency (88). Furthermore, constitutive activation of IKKβ appears to be sufficient to drive Treg development even in the absence of TCR ligation (194).

## NF-κB, Treg Homeostasis and Susceptibility to Autoimmunity

Once selected, ongoing NF-κB signals appear to be necessary to maintain Tregs and prevent end-organ inflammation. Comparison with Tconv reveals that there is greater accumulation of canonical NF-κB components in nuclei of Tregs (84). CD28 signals are important for Treg homeostasis in the periphery. Disruption of CD80/86 with CTLA4Ig results in a loss of Tregs (195, 196). T cell-intrinsic defects in the canonical pathway do not, however, always result in autoimmunity or organ-specific inflammation, as Tconv also exhibit a defect in activation.

Resting Tregs are predominantly located in secondary lymphoid organs due to specific chemokine receptor expression, while activated Tregs migrate into sites of inflammation and are characterized by a greater array of suppressive functions, including IL-10, Lag-3, TIGIT, CD73, and PD-1 expression (197). NF-κB also regulates Treg activation. Acquisition of the effector phenotype depends on TCR ligation (198). RelA appears to play a dominant role in maintaining effector Treg function in the periphery. *Rela* (p65) deletion is embryonic lethal, but recent evidence from conditional deficiency indicates that p65 is crucial for maintaining Treg lineage stability and activity (Table 1) (84, 85). Treg-specific p65 deficiency confers a multi-organ inflammatory phenotype. c-Rel deficiency confers a less prominent inflammatory phenotype (199). In addition to Treg homeostasis, canonical signaling appears to specify whether Tregs adopt either resting (CD62L^hi^ CD44^lo^) or activated/effector (CD62L^lo^ CD44^hi^) phenotypes (200).

## NF-κB Contributes to B Cell Homeostasis and Self-Tolerance

In mouse models, B cell survival depends on signals *via* BCR and BAFF (201–203), implicating signaling though canonical and non-canonical NF-κB pathways. Several mechanisms normally operate to eliminate autoreactive B cells (204). In the bone marrow, high affinity ligation by antigen results in receptor internalization, attenuation of NF-κB-mediated induction of Bcl-2, increased Bcl-2-interacting mediator (BIM), and reduced expression of regulation of B cell activating factor receptor (BAFFR). V(D)J recombination continues by virtue of ongoing RAG1/2 expression resulting in receptor editing but if immature B cells remain autoreactive they undergo clonal deletion. Receptor editing is marked by enhanced canonical signaling (205).

Clonal anergy represents a third mechanism by which autoreactive B cells enter the periphery with diminished capacity for activation. B cell anergy is characterized by reduced BCR expression, which attenuates canonical NF-κB survival signals and BAFFR expression is reduced, while pro-apoptotic signals such as BIM continue (206). BAFFR signals predominantly through the non-canonical pathway, and enhances B cell survival *via* induction of Bcl-2, PIM2, and PKCδ (207, 208). These actions reduce the risk of activation and survival of autoreactive B cells. BAFF is normally available in limiting amounts, which regulates the size of the transitional B cell compartment, but also regulates survival of autoreactive anergic B cells. Experimental manipulations demonstrate that excessive BAFF production results in improved survival of anergic B cells (206, 209).

## Rare Genetic Variants of NF-κB and Human Autoimmune Disease

Rare single gene disorders provide important evidence to support mechanistic pathways of human disease. Mutations affecting NF-κB activation, and its proximal signaling pathways, have so far been implicated overwhelmingly in susceptibility to infection (i.e., primary immune deficiency diseases). For the most part, these are loss-of-function mutations arising from homozygous or biallelic mutations. An exception is gain-of-function (GoF) mutations in *NFKBIA* (IκBα), but since this is the fundamental negative regulator of canonical signaling, GoF reinforces lack of activation and, therefore, results in a clinical and cellular phenotype that resembles LoF mutations in IKKγ and IKKβ (210, 211).

Several Mendelian human diseases nevertheless provide evidence that NF-κB protects against autoimmunity (Table 2). In other cases, NF-κB defects have been shown to cause auto-inflammatory diseases, which also result in tissue-specific inflammatory responses, but are distinguished by the absence of evidence for adaptive immune responses to autoantigens.

**Table 2:** Mendelian defects in NF-κB pathway genes that confer syndromes including autoimmune manifestations.

Gene	Protein	Inheritance	B cell and Ig phenotype	Regulatory T cell (Treg) phenotype	Autoimmunity or inflammation	Reference
*NFKB1*	p102/50	AD	Hypogammaglobulinemia	Reduced effector Tregs	Arthritis, pneumonitis, enteritis, ITP	(200, 212, 213)
*NFKB2*	p100/52	AD	Variable B cell deficiency; hypogammaglobulinemia	Reduced	Alopecia	(214–216)
*RELB*	RelB	AR	Memory B cell deficiency	ND	Arthritis, dermatitis	(217)
*MALT1*	MALT1	AR	Hypogammaglobulinemia	Reduced	Enteritis	(218)
*BCL10*	BCL-10	AR	Hypogammaglobulinemia	Reduced	Enteritis	(219)
*TNFAIP3*	A20	AD			Enteritis, dermatitis	(220, 221)
*OTULIN*	Otulin	AR			Enteritis, dermatitis	(97)

Syndromes arising from heterozygous mutations in *NFKB1* include autoimmune manifestations of arthritis, lung inflammation, gut inflammation, and immune-mediated thrombocytopenic purpura (ITP). There appears to be considerable clinical and cellular heterogeneity and postulated incomplete penetrance (212). The same mutation can also lead to antibody deficiency (213). Interestingly, as noted above, recent evidence from mouse studies showed that canonical signaling *via* RelA is important for maintaining Tregs in the periphery. Consistent with this, patients with *NFKB1* haploinsufficiency also show a defect in Tregs, with a diminution in effector Tregs (as judged by ICOS expression) (200).

*NFKB2* mutations result in a syndrome of antibody deficiency with variable B cell deficiencies (214–216). Patients with *NFKB2* mutations exhibit other clinical features as well, including defects of the anterior pituitary and alopecia. In at least some cases, the alopecia is reversible, consistent with alopecia areata. The fundamental defect in self-tolerance conferred by *NFKB2* mutations is yet to be resolved. Mouse models (outlined above) point to the possibility of a defect in central tolerance, and human *NFKB2* defects have been shown to confer a reduction in circulating Tregs (214). Whether there is a causal association between this observation and end-organ pathology remains to be resolved.

RelB deficiency conferred by a rare homozygous nonsense mutation has been reported in a single patient with severe polyarthritis and inflammatory skin disease, as well as immune deficiency (217). Interestingly, this phenotype was associated with enhanced nuclear p65 translocation, but a defect in T cell activation and thymus biopsy was reported to show a defect in T cell production.

MALT1 and BCL-10 deficiencies both appear to result in an IPEX (immune dysregulation polyendocrinopathy, enteropathy, X-linked; OMIM 304790)-like syndrome, which was originally identified in young boys with *FOXP3* deficiency and subsequently also observed with heterozygous mutations in *CTLA4* and *IL2RA* (CD25) (222–225). Homozygous *MALT1* mutations result in a phenotype of primary antibody deficiency together with enteropathy and a reduction in Tregs (218). A similar phenotype of enteropathy and Treg deficiency has been observed with *BCL10* deficiency (219). Again, the precise mechanism of action remains to be determined, although as noted above, CBM defects in mice are typically associated with Treg deficiency.

Several patients have been reported with a Mendelian syndromes arising from haploinsufficiency of *TNFAIP3* (220, 221). This autoinflammatory syndrome is reminiscent of Behcet’s disease, with manifestations, including aphthous ulceration, inflammatory bowel disease, and neutrophilic dermatoses. Biochemical analysis confirmed increased canonical pathway activation, prolonged nuclear translocation of p65, and enhanced transcription of NF-κB-dependent genes, including *IL6* and *TNF*. A similar autoinflammatory syndrome of panniculitis and dermatitis has been reported to arise from homozygous loss-of-function *OTULIN* mutations affecting the catalytic OTU domain. These mutations result in increased ubiquitination of several NF-κB proteins, including NEMO and RIPK1 (97).

## Polygenic Autoimmunity

The mechanisms explaining sporadic autoimmune disease remain poorly understood. Rare monogenic cases provide clues to pathogenic pathways that become dysregulated in more common versions of the disease, even if in any given individual these cellular and biochemical defects arise from more than one gene defect. Human *AIRE* mutations provide evidence for the importance of promiscuous thymic TSA expression in protecting against organ-specific autoimmune disease (160, 177, 226). Interestingly, autosomal-dominant defects in AIRE have now been reported, raising the possibility of more prevalent single gene causes of autoimmunity (227).

Thymic hyperplasia and thymoma have been associated with some forms of organ-specific autoimmunity, most notably, myasthenia gravis (MG), an autoimmune disease of the muscle end-plate. MG is characterized by muscle fatigability and weakness, is often associated with thymic hyperplasia or thymoma, together with defects in CD4^+^ T cell selection and intrathymic cytokine production. Thymectomy is sometimes curative (228). Certain common *AIRE* polymorphisms segregate with MG, particularly in early-onset cases (229).

While plausible, there is no empirical evidence that genetic variants affecting non-canonical NF-κB signaling segregate with organ-specific autoimmune disease. The most consistent NF-κB genetic association with autoimmunity is *TNFAIP3*. Numerous coding and non-coding polymorphisms have been shown to segregate with autoimmune diseases, including lupus and organ-specific autoimmune diseases (230–232). Polymorphisms of *REL* (c-Rel) have been shown to segregate with both psoriatic arthritis and rheumatoid arthritis, and altered expression has been noted in other autoimmune diseases (233–236). Common *NFKB1* polymorphisms have been shown to segregate with several autoimmune and inflammatory diseases (237–239). More recently, a polymorphism within the *NFKB1* locus was shown to segregate with multiple sclerosis. Interestingly, MS patients were found to have increased levels of p65 (RelA) activation, and the disease associated polymorphism was found to be associated with increased expression of BCL-3, TNFAIP3, and CIAP1 (240). This represents important progress toward understanding how common variants in NF-κB might contribute to sporadic and more common autoimmune diseases, *via* pathways now well understood from investigations of animal models and patients with rare monogenic disease.

## Conclusion

NF-κB is well established as a regulator of innate and adaptive mechanisms of host defense, and specific genetic defects that confer immune deficiency confirm the importance of these mechanisms. As described here, there is also abundant evidence that NF-κB is crucial for maintaining immunological tolerance, as a result of its actions during thymic selection, both for negative selection of autoreactive T cells, and selection and maintenance of Tregs. The non-canonical NF-κB pathway appears to be particularly important for normal mTec function; nevertheless, evidence has also emerged for a significant lymphocyte-intrinsic action of the canonical pathway for maintaining T cell tolerance. Further work is required to accomplish the challenging task of separating the actions of canonical and non-canonical components, cell-intrinsic versus cell extrinsic actions, actions on negative selection versus Treg defects. Finally, it is important to note that since NF-κB is crucial for Tconv function in the periphery, it is possible that defects in selection may be offset by defects in the Tconv compartment.

## Author Contributions

Both the authors contributed to conceptualizing and writing the manuscript.

## Conflict of Interest Statement

The authors declare that the research was conducted in the absence of any commercial or financial relationships that could be construed as a potential conflict of interest.

## Abbreviations

Aire, autoimmune regulator; BAFFR, regulation of B cell activating factor receptor; BIM, Bcl-2-interacting mediator; CBM, CARD/BCL-10/MALT1 complex; cTEC, cortical thymic epithelial cell; DC, dendritic cell; DUB, deubiquitinase; EAE, experimental autoimmune encephalomyelitis; ENU, *N*-ethyl-*N*-nitrosourea; Ep-CAM, epithelial cellular adhesion molecule; GC, germinal center; IκB, inhibitor of kappa B; IKK, inhibitor of kappa B kinase; LPS, lipopolysaccharide; mTEC, medullary thymic epithelial cell; MZ, marginal zone; NEMO, NF-κB essential modulator; NF-κB, nuclear factor kappa-light-chain-enhancer of activated B cells; NIK, NF-κB-inducing kinase; nTreg, natural regulatory T cell; RANK, receptor activator of nuclear factor kappa-B; TAD, transactivation domain; Tconv, Conventional T cell; Treg, regulatory T cell; TSA, tissue specific antigens; UEA-1, *Ulex europaeus* agglutinin 1.
